# Significance of inflammatory indexes in atezolizumab monotherapy outcomes in previously treated non-small-cell lung cancer patients

**DOI:** 10.1038/s41598-020-74573-0

**Published:** 2020-10-15

**Authors:** Yuki Katayama, Tadaaki Yamada, Yusuke Chihara, Satomi Tanaka, Keiko Tanimura, Naoko Okura, Kazuki Hirose, Sayaka Uda, Shinsuke Shiotsu, Soichi Hirai, Osamu Hiranuma, Taishi Harada, Takayuki Shimamoto, Masahiro Iwasaku, Yoshiko Kaneko, Junji Uchino, Takayuki Takeda, Koichi Takayama

**Affiliations:** 1 Department of Pulmonary Medicine, Graduate School of Medical Science, Kyoto Prefectural University of Medicine, 465, Kajii-cho, Kamigyo-ku, Kyoto, 602–8566 Japan; 2 Department of Respiratory Medicine, Uji-Tokushukai Medical Center, Uji, Japan; 3 Department of Respiratory Medicine, Japanese Red Cross Kyoto Daini Hospital, Kyoto, Japan; 4 Department of Respiratory Medicine, Japanese Red Cross Kyoto Daiichi Hospital, Kyoto, Japan; 5 Department of Respiratory Medicine, Otsu City Hospital, Otsu, Japan; 6 Department of Medical Oncology, Fukuchiyama City Hospital, Fukuchiyama, Japan

**Keywords:** Cancer, Immunology, Biomarkers, Oncology

## Abstract

Cancer immunotherapy, including atezolizumab monotherapy, is a promising alternative strategy for patients with advanced non-small-cell lung cancer (NSCLC). Several inflammatory indices have been reported as potential biomarkers regarding the effectiveness of various treatments. This study aimed to analyze the efficacy of atezolizumab monotherapy using baseline inflammatory markers in NSCLC patients. We retrospectively enrolled 81 NSCLC patients who received atezolizumab monotherapy at six different medical institutions in Japan. The Cox proportional hazards model was used to assess the impact of the clinical variables, including inflammatory indexes, on clinical outcomes. Median progression-free survival (PFS) and overall survival (OS) were 60 days and 252 days, respectively. The objective response rate was 7.4%, and the disease control rate was 54.3%. Patients with high neutrophil to lymphocyte ratio (NLR), low lymphocyte to monocyte ratio (LMR), and/or high platelet to lymphocyte ratio (PLR), at baseline, demonstrated substantially shorter PFS and OS compared to those with a low NLR, high LMR, and/or low PLR. The multivariate analysis demonstrated that a high baseline NLR was substantially associated with short PFS and short OS. Our retrospective observations suggest that inflammatory indices may be a potential negative prognostic factor of atezolizumab monotherapy outcomes in NSCLC patients.

## Introduction

Lung cancer is the leading cause of cancer death worldwide^1^. Immune checkpoint inhibitors (ICIs), which target the programmed cell death protein 1 (PD-1) and its ligand, programmed death-ligand 1 (PD-L1), have been approved in the United States, Japan, and other countries, for the treatment of non-small-cell lung cancer (NSCLC) patients. The PD-1 receptor is expressed on activated T cells and binds to PD-L1 and PD-L2 to avoid autoimmunity in peripheral tissues^2^. Clinically, the difference in blockade of PD-1 and PD-L1 is interesting. Treatment with monoclonal antibodies specific for PD-L1 can still permit binding between PD-1 and PD-L2, and result in reduced blockade of the negative inhibitory signals of the immune system in comparison to PD-1 antibodies. Further, a systematic review has demonstrated that PD-L1 inhibitors have a slightly lower incidence of grade 3/4 immune-related pneumonitis as compared to PD-1 inhibitors^3^, while anti-PD-1 and anti-PD-L1 antibody monotherapy shows a similar clinical response in previously treated NSCLC patients^4–7^. Hence, investigations into the clinical biomarkers of effective anti-PD-L1 antibody treatment, which is a promising therapeutic strategy for NSCLC, are warranted.

Atezolizumab is a humanized, engineered monoclonal antibody that targets PD-L1, and contributes to preventing the interaction between PD-L1 and B7.1 receptor. The OAK study, a randomized phase 3 trial, demonstrated that the atezolizumab treatment group had a median OS of 13.8 months, which was substantially higher than the 9.9 months observed for the docetaxel group. Moreover, atezolizumab monotherapy showed tolerability with a more favorable safety profile than docetaxel^3,4^. PD-L1 expression in tumors has been used clinically as a positive biomarker for effective ICI treatment in NSCLC^5^. However, the anti-PD-L1 antibody clone SP142, which was utilized for clinical trials with atezolizumab, was relatively less concordant in PD-L1 expression than other antibodies, such as 28–8, 22C3, and SP263 in patients with NSCLC^6,7^. Furthermore, several recent studies have reported potential ICI biomarkers in the host, such as preexisting autoimmune antibodies^8^, steroid use^9^, microbiome^10^, white blood cell count^11^, sarcopenia^12^, and body mass index (BMI)^13–15^. Several inflammatory indices, such as the NLR, LMR, and PLR, which are recognized as important markers of inflammatory processes, have also been reported as potential predictors of the effectiveness of anti-PD-1 antibody therapy^16–18^. However, little is known regarding the subpopulation of NSCLC patients who exhibit clinical outcomes that require treatment with atezolizumab monotherapy. In this retrospective study, we analyzed the efficacy of atezolizumab monotherapy, using the baseline values of specific inflammatory markers, in 81 patients previously treated for NSCLC.

## Results

### Patient characteristics

A total of 81 NSCLC patients, treated with atezolizumab between April 2018 and November 2019 at six different medical institutions in Japan, were enrolled in this study. The sample characteristics included a median age of 71 years (range 42–84), with 44 male patients (54.3%), and 64 (79.0%) patients with a history of smoking. The histological subtypes were 50 adenocarcinoma (61.7%) and 17 squamous cell carcinomas (21.0%). Metastatic disease was detected in the liver of 11 patients (13.6%) and in the brain of 22 patients (27.2%). With regards to disease staging, 19 patients (23.5%) were at stage III, 51 (63.0%) were at stage IV, and 11 (13.6%) showed postoperative recurrence. An EGFR mutation was detected in 14 patients (17.3%). There were no ALK positive patients. The ECOG-PS was 0–1 for 64 of the patients (79.0%) and 2–4 for 17 patients (21.0%). The PD-L1 TPS was ≥ 50% in 13 patients (16.0%), 1–49% in 24 patients (29.6%), and < 0% in 28 patients (34.6%) with 16 patients non-evaluable (19.8%). The BMI was ≥ 25 for 10 patients (12.3%), 20–25 for 40 patients (49.4%), and < 20 for 31 patients (38.3%). Furthermore, 14 patients (17.2%) experienced an irAE of any grade. Atezolizumab treatment was administered as 2nd line therapy for 14 patients (17.2%), 3rd line for 22 patients (27.2%), and 4th line or later for 45 patients (55.6%). Table 1 details the baseline characteristics of the patients enrolled in this study.

**Table 1:** Patient characteristics at the baseline.

Items	Group	n (%)
Age	Median (range)	71 (42–84)
Gender	Male	44 (54.3)
	Female	37 (45.7)
ECOG-PS	0	23 (28.4)
	1	41 (50.6)
	2	10 (12.3)
	3	7 (8.6)
Histology	Adenocarcinoma	50 (61.7)
	Squamous cell carcinoma	17 (21.0)
	Other	14 (17.3)
Smoking status	Never smoker	17 (21.0)
	Current or former smoker	64 (79.0)
Staging	Stage III	19 (23.5)
	Stage IV	51 (63.0)
	Postoperative recurrence	11(13.6)
EGFR mutations	Positive	14 (17.3)
	Negative	67 (82.7)
PD-L1 TPS	≥ 50%	13 (16.0)
	1–49%	24 (29.6)
	< 1%	28 (34.6)
	Not evaluation	16 (19.8)
Metastasis	Liver metastasis	11 (13.6)
	Brain metastasis	22 (27.2)
BMI	BMI > 25	10 (12.3)
	25 ≥ BMI > 20	40 (49.4)
	BMI ≤ 20	31 (38.3)
Immune-related adverse events (irAE)	Yes	14 (17.3)
	No	67 (82.7)
Treatment line	2nd	14 (17.3)
	3rd	22 (27.2)
	≥ 4th	45 (55.6)

### The roles of inflammatory indexes in atezolizumab treatment

Based on the RECIST criteria, with regards to atezolizumab treatment outcomes, no patients experienced a complete response (0%), 6 experienced a partial response (7.4%), 38 were classified with a stable disease (46.9%), 28 met the criteria for progressive disease (34.6%), and 9 were non-evaluable (11.1%). The objective response rate was 7.4% (95% CI 2.8%–15.4%), and the disease control rate was 54.3% (95% CI 42.9%–65.4%). The median PFS and OS were 60 days (95% CI 49–86 days) and 252 days (95% CI 197–NA days), respectively.

**Figure 1:**
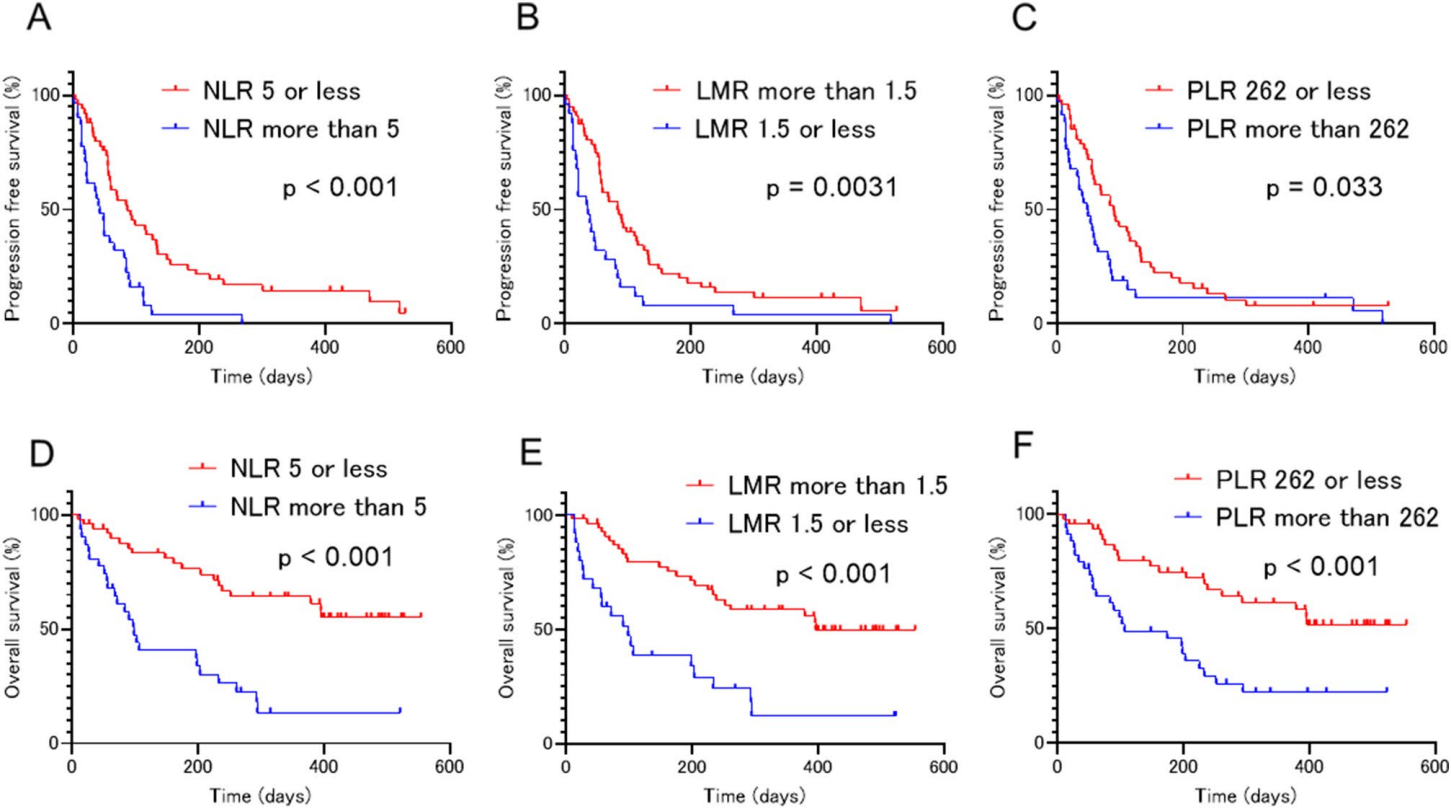
Kaplan–Meier survival curves for progression-free survival (PFS) and overall survival (OS). (**A**) The neutrophil to lymphocyte ratio (NLR) > 5 (42 days vs. 86 days; p < 0.001), (**B**) the lymphocyte to monocyte ratio (LMR) ≤ 1.5 (37 days vs. 84 days; p = 0.0031), and (**C**) the platelet to lymphocyte ratio (PLR) > 262 (48.5 days vs. 90 days; p = 0.033) were significantly associated with shorter PFS. (**D**) The neutrophil to lymphocyte ratio (NLR) > 5 (98 days vs. NA; p < 0.001), (**E**) the lymphocyte to monocyte ratio (LMR) ≤ 1.5 (98 days vs. 396 days; p < 0.001), and (**F**) the platelet to lymphocyte ratio (PLR) > 262 (106 days vs. NA; p < 0.001) were significantly associated with shorter OS.

According to the log-rank test, patients with an NLR > 5, LMR ≤ 1.5, and PLR > 262 demonstrated significantly shorter PFS than those with an NLR ≤ 5, LMR > 1.5, and PLR ≤ 262, respectively (42 days vs. 86 days, p < 0.001; 37 days vs. 84 days, p = 0.0031; 48.5 days vs. 90 days, p = 0.033, respectively) (Fig. 1A–C). Additionally, patients with an NLR > 5, LMR ≤ 1.5, and PLR > 262 exhibited significantly shorter OS than those with an NLR ≤ 5, LMR > 1.5, and PLR ≤ 262, respectively (98 days vs. NA, p < 0.001; 98 days vs. 396 days, p < 0.001; 106 days vs. NA, p < 0.001, respectively) (Fig. 1D–F). Additionally, univariate analysis revealed that a neutrophil count > 4500/mm^3^ (hazard ratio (HR): 1.62; 95% CI 1.01–2.59; p = 0.042), lymphocyte count > 1000/mm^3^ (HR: 0.60; 95% CI 0.38–0.96; p = 0.033), NLR > 5 (HR: 2.47; 95% CI 1.50–4.06: p < 0.001), LMR ≤ 1.5 (HR: 0.48; 95% CI 0.30–0.79; p = 0.0040), and PMR > 262 (HR: 1.67; 95% CI 1.04–2.68; p = 0.035) were significantly associated with PFS in patients receiving atezolizumab treatment. Further, univariate analysis of the patient data also revealed that neutrophil counts > 4500/mm^3^ (HR: 2.56; 95% CI 1.38–4.74; p = 0.0028), lymphocyte counts > 1000/mm^3^ (HR: 0.47; 95% CI 0.26–0.87; p = 0.015), monocyte counts > 500/mm^3^ (HR: 1.96; 95% CI 1.07–3.57; p = 0.029), NLR > 5 (HR: 3.78; 95% CI 2.04–7.04; p < 0.001), LMR ≤ 1.5 (HR: 0.30; 95% CI 0.17–0.55: p < 0.001), and PLR > 262 (HR: 2.82; 95% CI 1.54–5.18; p < 0.001) were significantly associated with OS (Table 2).

**Table 2:** Cox proportional hazards and logistic regression models for progression free survival (PFS) and overall survival (OS).

Items	PFS (univariate analysis)		OS (univariate analysis)	
	HR (95% CI)	p-value	HR (95% CI)	p-value
Age ≥ 75 years	0.74 (0.43–1.25)	0.25	1.14 (0.60–2.15)	0.69
Male gender	1.76 (1.08–2.85)	0.022	1.48 (0.81–2.71)	0.20
Smoker	1.02 (0.58–1.81)	0.94	1.59 (0.71–3.57)	0.26
ECOG-PS ≥ 2	1.39 (0.78–2.46)	0.27	1.92 (0.99–3.74)	0.054
Squamous histology	0.99 (0.56–1.73)	0.96	0.79 (0.41–1.53)	0.49
EGFR mutations positive	1.52 (0.83–2.78)	0.18	1.24 (0.55–2.79)	0.61
Treatment line ≥ 4th	1.11 (0.70–1.77)	0.66	1.84 (0.99–3.41)	0.053
BMI > 20	0.76 (0.47–1.22)	0.25	0.47 (0.26–0.85)	0.012
BMI > 25	0.88 (0.42–1.85)	0.74	0.56 (0.20–1.56)	0.26
Alb > 3.8 g/dL	0.68 (0.41–1.13)	0.136	0.52 (0.27–1.01)	0.0547
CRP > 0.89 mg/dL	1.33 (0.83–2.11)	0.23	2.36 (1.27–4.37)	0.0064
LDH > 227 U/L	1.59 (1.00–2.54)	0.052	1.67 (0.91–3.04)	0.095
Neutrophil > 4500/mm^3^	1.62 (1.01–2.59)	0.042	2.56 (1.38–4.74)	0.0028
Lymphocyte > 1000/mm^3^	0.60 (0.38–0.96)	0.033	0.47 (0.26–0.87)	0.015
Monocyte > 500/mm^3^	1.53 (0.96–2.44)	0.072	1.96 (1.07–3.57)	0.029
Platelet > 250,000/mm^3^	1.29 (0.80–2.08)	0.29	1.31 (0.72–2.38)	0.37
NLR > 5.0	2.47 (1.50–4.06)	< 0.001	3.78 (2.04–7.04)	< 0.001
LMR > 1.5	0.48 (0.30–0.79)	0.0040	0.30 (0.17- 0.55)	< 0.001
PLR > 262	1.67 (1.04–2.68)	0.035	2.82 (1.54–5.18)	< 0.001
Liver metastasis	1.85 (0.96–3.54)	0.064	1.55 (0.69–3.48)	0.29
Brain metastasis	1.26 (0.75–2.09)	0.38	1.60 (0.85–3.02)	0.15
irAE	1.81 (0.92–3.56)	0.09	1.23 (0.52–2.91)	0.64
PD-L1 TPS 1–49% (vs. < 1%)	1.46 (0.86–2.49)	0.16	1.31 (0.65–2.64)	0.46
PD-L1 TPS ≥ 50% (vs. 0–49%)	1.26 (0.66–2.40)	0.48	2.02 (0.93–4.37)	0.076

Multivariate analysis included age, ECOG-PS, smoking history, NLR, albumin (Alb), and C-reactive protein (CRP), while excluding LMR, PLR, neutrophil count, and lymphocyte count to avoid multi-collinearity among the NLR, LMR, and PLR. Our multivariate analysis demonstrated that a high baseline NLR was independently associated with PFS (HR: 2.50; 95% CI 1.40–4.56: p = 0.0018) and OS (HR: 2.91: 95% CI 1.51–5.61; p = 0.0014) in patients receiving atezolizumab treatment (Table 3).

**Table 3:** Cox proportional hazards and logistic regression models for progression free survival (PFS) and overall survival (OS) including the neutrophil to lymphocyte ratio (NLR).

Items	PFS (multivariate analysis)		OS (multivariate analysis)	
	HR (95%CI)	*p*-value	HR (95%CI)	*p*-value
Age > 75 years	0.66 (0.37–1.15)	0.14	1.10 (0.57–2.12)	0.78
ECOG-PS ≥ 2	1.39 (0.75–2.58)	0.073	1.63 (0.81–3.30)	0.17
Smoker	0.98 (0.54–1.80)	0.93	1.28 (0.56–2.92)	0.56
NLR > 5.0	2.50 (1.40–4.56)	0.0018	2.91 (1.51–5.61)	0.0014
Alb > 3.8 g/dL	0.92 (0.53–1.60)	0.76	0.80 (0.40–1.62)	0.54
CRP > 0.89 mg/dL	0.97 (0.56–1.70)	0.92	1.63 (0.83–3.19)	0.16

### Clinical profiles associated with baseline NLR

**Figure 2:**
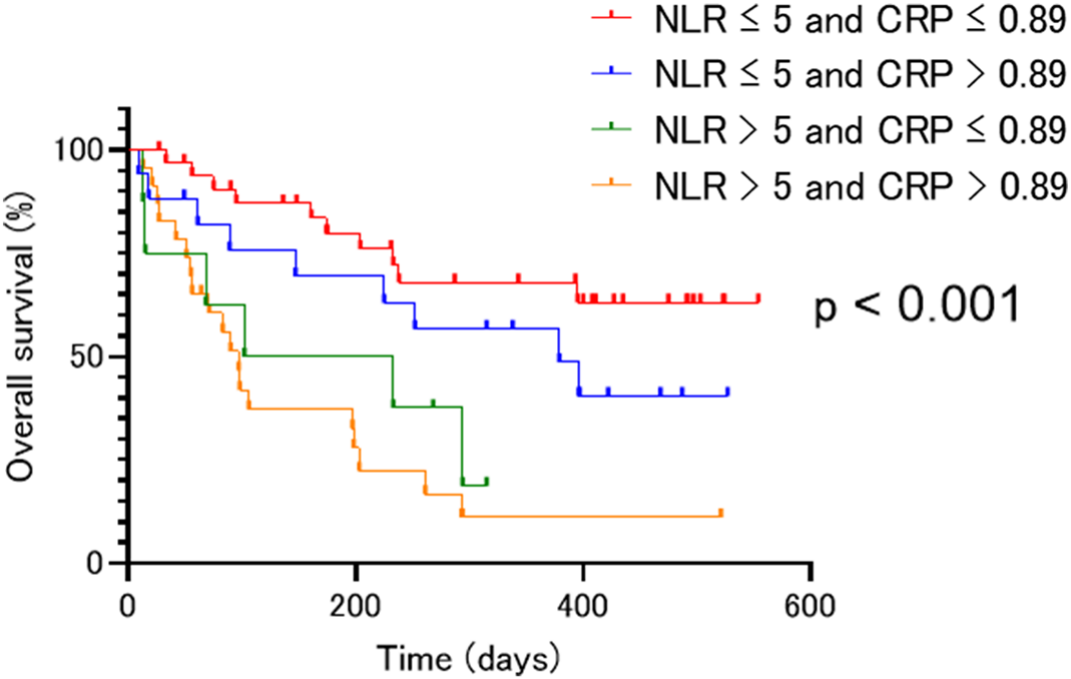
Kaplan–Meier survival curves for overall survival (OS) according to the combination of NLR and CRP levels. Overall survival (OS) was substantially longer in the group with a NLR ≤ 5 and CRP ≤ 0.89 mg/L (NA) in comparison to the other groups with a NLR ≤ 5 and CRP > 0.89 mg/L (379 days), an NLR > 5 and CRP ≤ 0.89 mg/L (167.5 days), and an NLR > 5 and CRP > 0.89 mg/L (97 days) (p < 0.001).

Of the 81 patients, 31 (38.3%) had a pretreatment NLR of > 5 and the remaining 50 patients (61.7%) had a pretreatment NLR of ≤ 5. When comparing the clinical profiles of these two groups (Table 4), the continuous variables BMI, CRP, and Alb were found to be substantial prognostic factors in patients with pretreatment NLR > 5 (p = 0.021, p = 0.0015, and p < 0.001, respectively). According to the log-rank test, an additional analysis showed that the combination of NLR and CRP was substantially correlated with OS, which indicates the synergistic effect of the combined use of NLR and CRP as prognostic factors in NSCLC patients receiving atezolizumab monotherapy (Fig. 2).

**Table 4:** Patient characteristics related to baseline neutrophil to lymphocyte ratio (NLR) (n = 81).

Items	NLR > 5 (n = 31)	NLR ≤ 5 (n = 50)	p value
**Age**			
Median (range)	71 (47–84)	71 (42–82)	0.71
**Gender**			
Male	20	24	0.17
Female	11	26	
**Smoking status**			
Smoker	26	38	0.58
Non-smoker	5	12	
**ECOG-PS**			
0–1	23	41	0.42
2–4	8	9	
**Histology**			
Sq	8	10	0.59
non-Sq	23	40	
**EGFR mutation status**			
Positive	6	8	0.14
Negative	25	42	
**BMI**			
Median (range)	19.2 (12.7–26.6)	21.3 (14.7–24.4)	0.021
**Alb**			
Median (range)	3.40 (2.0–4.7)	3.84 (2.0–4.6)	0.0015
**CRP**			
Median (range)	3.06 (0.16–28.64)	0.26 (0.01–10.46)	< 0.001
**LDH**			
Median (range)	250 (156–794)	223 (143–1442)	0.472
**Liver metastasis**			
Positive	5	6	0.741
Negative	26	44	
**Brain metastasis**			
Positive	11	11	0.21
Negative	20	39

## Discussion

Several studies have demonstrated that clinical characteristics, such as age, ECOG‐PS, and smoking status, are negative biomarkers related to the clinical outcomes of anti-PD-1 antibody treatment in patients with NSCLC^16,17,19^. In contrast, current clinically useful biomarkers have not been fully identified in predicting the efficacy of anti-PD-L1 antibody atezolizumab monotherapy. A pooled cohort analysis of clinical trials involving 1,489 NSCLC patients demonstrated the significance of the lung immune prognostic index, which is derived from scoring of the baseline LDH levels and NLR, in predicting survival and response outcomes of NSCLC patients treated with atezolizumab^20^. In the current retrospective analysis of 81 NSCLC patients who received atezolizumab monotherapy, the pretreatment inflammatory indices, more specifically high NLR, low LMR, and high PLR, were substantially associated with shorter PFS and OS.

Increasing evidence suggests that cancer-related inflammation plays an important role in tumor development. Peripheral blood leukocytes, including neutrophils and lymphocytes, are involved in the systemic inflammatory response, and participate in tumorigenesis and tumor progression. Previous studies have shown that high levels of neutrophils promote cancer cell proliferation, invasion, and metastasis, and induce resistance to cancer therapeutics^21,22^. Additionally, peripheral neutrophil counts reportedly correlate directly with the intratumoral neutrophil population^23^. In contrast, lymphocytes inhibit tumor growth and invasion through their cytolytic activity. In fact, the immune response to human cancer cells depends primarily on the level of total lymphocytes, which can be sharply reduced by systemic inflammation. Specifically, relative lymphocytopenia may reflect lower levels of CD4 + T cells, which impairs cancer immune surveillance and defense^21,24^.

NLR is a marker of the systemic inflammatory response and reflects the balance between neutrophils and lymphocytes^25,26^. Pretreatment NLR is associated with the clinical outcomes of several therapeutic interventions in NSCLC patients, such as the response to platinum-based first-line chemotherapy in metastatic NSCLC patients, and the prognosis in operable NSCLC patients^27,28^. Moreover, inflammatory indices, including NLR, PLR, and LMR, are potential prognostic markers for lung cancer patients^29–32^. Our multivariate analysis demonstrated that high baseline NLR is an independent factor associated with poor PFS and OS. It is; however, unclear whether a high NLR is an effective prognostic factor in NSCLC patients receiving atezolizumab monotherapy. Therefore, it is necessary to further evaluate the clinical role of inflammatory scores, including NLR, in future studies.

As several independent clinical factors have indicated the disadvantages of immunotherapy in NSCLC patients, we evaluated the combined scores of several clinical characteristics. Our findings revealed that patients with high NLR are substantially correlated with several other clinical characteristics, such as low BMI, low Alb, and high CRP levels, at the baseline, compared to those with a low NLR. The present study is the first to reveal that the combination of a high baseline NLR and CRP levels is a potent prognostic factor for NSCLC patients receiving atezolizumab treatment. Moreover, persistent inflammatory responses in tumors suppress anti-tumor immunity and promote cancer progress through several mechanisms, including activation of type 2 T helper responses, which recruits regulatory T cells, and activation of the chemokine system^33,34^. Subsequently, neutrophils can be induced by cancer-associated inflammatory chemokines and cytokines, such as interleukin (IL)-6 and tumor necrosis factor^35,36^. CRP is also regulated by IL-6 and IL-1β, suggesting that induction of neutrophils and CRP may occur via similar inflammatory pathways. In fact, our results highlight an important relationship between high NLR and high CRP levels and promotion of poor prognosis. Although the usefulness of these combinatorial indexes remains largely unknown, they have the potential to serve as accurate biomarkers of cancer-related inflammation; hence, further large-scale investigations are warranted.

High plasma tumor mutational burden has been identified as a pivotal biomarker for the efficacy of atezolizumab monotherapy, and is associated with superior PFS in patients with previously treated NSCLC^37^; however, its associated cost makes in unfeasible for daily use. Alternatively, blood count analysis and CRP assessments are cost effective, form part of routine clinical practice, and reveal prognostic factors that could be useful in identifying NSCLC patients who will respond poorly to atezolizumab monotherapy, thereby assisting clinical decision-making regarding appropriate therapeutic interventions in previously treated NSCLC.

The present study has several limitations. Firstly, it is a retrospective study and the cohort had a limited sample size of 81 cases, even though treatment was administered in multiple medical institutions. Secondly, all patients in the cohort were Japanese. Thirdly, the study included several biases regarding patient conditions at commencement of atezolizumab therapy, such as the number of pretreatment regimens and the ECOG-PS of the patients. Finally, our findings revealed a substantial relationship between pre-treatment blood inflammatory markers and clinical outcomes, such as PFS and OS, in NSCLC patients treated with atezolizumab. These inflammatory markers might be a prognostic factor rather than a predictive factor for patients with this disease treated with atezolizumab. Although the current study was retrospective in nature, our novel biomarker findings regarding patient response to atezolizumab are notable and could be useful in addressing clinical issues. Future prospective investigations are necessary to verify our findings.

In summary, our observations showed that pretreatment inflammatory indexes, including a high NLR, could be promising negative prognostic factors for atezolizumab treatment in patients with previously treated for NSCLC. Since this retrospective study was conducted on a smaller scale, further experiments are needed to validate these observations.

## Methods

### Patients

We enrolled 81 patients, previously treated with chemotherapy for advanced NSCLC, who initiated atezolizumab monotherapy. The patients were treated between April 2018 and November 2019 at six different medical institutions, namely University Hospital, Kyoto Prefectural University of Medicine (Kyoto, Japan), Japanese Red Cross Kyoto Daiichi Hospital (Kyoto, Japan), Japanese Red Cross Kyoto Daini Hospital (Kyoto, Japan), Uji‐Tokushukai Medical Center (Kyoto, Japan), Fukuchiyama City Hospital (Kyoto, Japan), and Otsu City Hospital (Shiga, Japan).

Atezolizumab was intravenously administered to patients as a fixed dose of 1200 mg every three weeks. In general, these treatments continued until disease progression, intolerable toxicity, or patient refusal was noted. By the follow-up date, 72 (88.9%) of the 81 patients had experienced progression of the disease, including 44 (54.3%) that had passed away, while 37 patients (45.7%) survived. The patients’ clinical data, including age, sex, height, weight, BMI at the start of atezolizumab administration, histological subtype, PD‐L1 expression level in tumors, epidermal growth factor receptor (EGFR) mutation status, anaplastic lymphoma kinase (ALK) fusion status, disease staging, metastatic site, corticosteroid administration, Eastern Cooperative Oncology Group Performance Status (ECOG‐PS), smoking status, baseline laboratory findings, OS, PFS, as well as response rate and disease control rate, based on the Response Evaluation Criteria in Solid Tumors (RECIST; version 1.1), were retrospectively obtained from their medical records. The tumor–node–metastasis (TNM) stage was classified using the TNM stage classification system, version 8. The study protocol was approved by the Ethics Committees of the Kyoto Prefectural University of Medicine and of each hospital. The work described herein has been carried out in accordance with the principles of the Declaration of Helsinki. Informed consent was obtained from all participants.

### Tumor PD-L1 analysis

PD-L1 expression was analyzed by SRL, Inc. using a PD-L1 IHC 22C3 pharmDx assay (Agilent Technologies, Santa Clara, CA). The PD-L1 tumor proportion score (TPS) was calculated as a percentage in at least 100 viable tumor cells with complete, or partial, membrane staining. Pathologists at SRL, Inc. interpreted the TPS results.

### Laboratory findings

The NLR, LMR, and PLR were defined as absolute neutrophil counts divided by absolute lymphocyte count, absolute lymphocyte count divided by absolute monocyte count, and absolute platelet count divided by absolute lymphocyte count, respectively. We measured baseline albumin (Alb), C-reactive protein (CRP), lactate dehydrogenase (LDH), as well as neutrophil, lymphocyte, monocyte, and platelet counts, and NLR, LMR, and PLR. Baseline was defined as day − 10 to 0 of the first atezolizumab administration. Cut off points of NLR = 5, LMR = 1.5, and PLR = 262 were selected based on previous studies^16,19^. The cutoff values for baseline albumin, LDH, CRP, neutrophil, lymphocyte, monocyte, and platelets, were the respective median values.

### Statistical analysis

Statistical analyses were performed using EZR statistical software, version 1.30^38^. All statistical tests carried out were two-sided and p < 0.05 was regarded as statistically significant. The PFS and OS were calculated using the Kaplan–Meier method, and differences were compared using the log-rank test. Continuous variables were analyzed using the Mann–Whitney U test, while categorical variables were analyzed using Fisher's exact test. Univariate analyses were performed using the Cox proportional hazards and logistic regression models.

## Data Availability

The datasets generated during and/or analysed during the current study are available from the corresponding author on reasonable request.
